# An Unusual Presentation of Cryoglobulinemia in a Patient With Undiagnosed Sjögren’s Syndrome and Treated Marginal Zone Lymphoma

**DOI:** 10.7759/cureus.32730

**Published:** 2022-12-20

**Authors:** Jacob Patrick, Abida Babu, Neha Verma

**Affiliations:** 1 Internal Medicine, University of South Florida Morsani College of Medicine, Tampa, USA; 2 Internal Medicine, Moffitt Cancer Center, Tampa, USA

**Keywords:** sjögren’s syndrome, grave’s disease, vasculitis, cryoglobulinemia, mantle cell lymphoma

## Abstract

Our case details a 47-year-old female who presented to our cancer hospital with a petechial rash of the lower extremities as well as a headache and blurred vision for the prior two days. She was found to have systolic pressures in the 200s in the emergency department and was admitted for a hypertensive emergency. Notable medical history includes marginal zone lymphoma (MZL) status post-submandibular resection at an outside institution in 11/2017 (thought to be in remission). With her history in mind, she also reported subjective submandibular swelling on admission and an unintentional 25-pound weight loss over the eight weeks prior to admission. A PET scan was completed, which showed diffusely increased reticuloendothelial activity, and a follow-up bone marrow biopsy was without residual lymphoma activity. Creatinine was markedly elevated with significant proteinuria, and a renal biopsy revealed thrombotic microangiopathy, acute tubular injury, and moderate interstitial fibrosis. Remarkable laboratory tests included positive quantitative cryoglobulins (“cryocrit”) and low complement 4 (C4). Qualitative cryoglobulins were never obtained, unfortunately. She was started on prednisone and transferred to a nearby academic hospital for formal rheumatologic evaluation. Importantly, testing at this facility showed elevated Sjögren’s syndrome-related antigen A (SSA/Ro) antibodies. Also elicited at the academic hospital was that she had been experiencing symptoms of chronic dry eyes and mouth years even before her diagnosis of MZL. She was diagnosed with primary Sjögren’s syndrome, which was thought to be the cause of her likely mixed cryoglobulinemia and the precipitant of her acute renal failure with hypertensive emergency, her skin changes, her anemia, and her hypocomplementemia. Of note, prior to discharge from the academic hospital, the patient’s cryoglobulin testing was negative after prolonged steroid treatment, and she was placed on rituximab for maintenance. Our case is important as it helps illustrate one of the myriad precipitants of mixed cryoglobulinemia, in this case possibly untreated Sjögren’s syndrome.

## Introduction

Cryoglobulinemia can be caused by a variety of etiologies including hepatitis C (chiefly), lymphoproliferative disease, and autoimmune disease. Cryoglobulins are immunoglobulins known to precipitate at low temperatures, which classically lead to systemic inflammation manifesting as glomerulonephritis, arthralgia, fatigue, and neuropathy, among others. Manifestations result from the deposition of immune complexes in small- to medium-sized blood vessels [1]. Diagnosis is based on manifestations of clinical disease and positive testing for serum cryoglobulins (cryocrit) [2]. Our case is unique as it demonstrates an uncommon cause of cryoglobulinemia, primary Sjögren’s syndrome. About 3%-4% of patients with primary Sjögren’s syndrome may go on to develop cryoglobulinemic vasculitis, and it is associated with a relatively high risk of B-cell lymphoma, the details of which will be discussed later [3].

## Case presentation

Our case details a 47-year-old female who presented to our cancer hospital with a petechial rash of the lower extremities as well as headache and blurred vision for the prior two days. She was found to have systolic pressures in the 200s in the emergency department and was admitted for a hypertensive emergency. She had recently been placed on a blood pressure regimen of three medications (amlodipine, metoprolol, and lisinopril) by an outside physician but self-discontinued as she attributed them to the development of the petechial rash. Notable medical history includes marginal zone lymphoma (MZL) status post-submandibular resection at an outside institution in 11/2017 (thought to be in remission) and Grave’s disease status post-thyroidectomy in 2013. With her history in mind, she also reported subjective submandibular swelling on admission as well as an unintentional 25-pound weight loss over the eight weeks prior to admission. A PET scan was completed, which showed diffusely increased reticuloendothelial activity; a follow-up bone marrow biopsy fortunately revealed no residual lymphoma activity.

**Figure 1:**
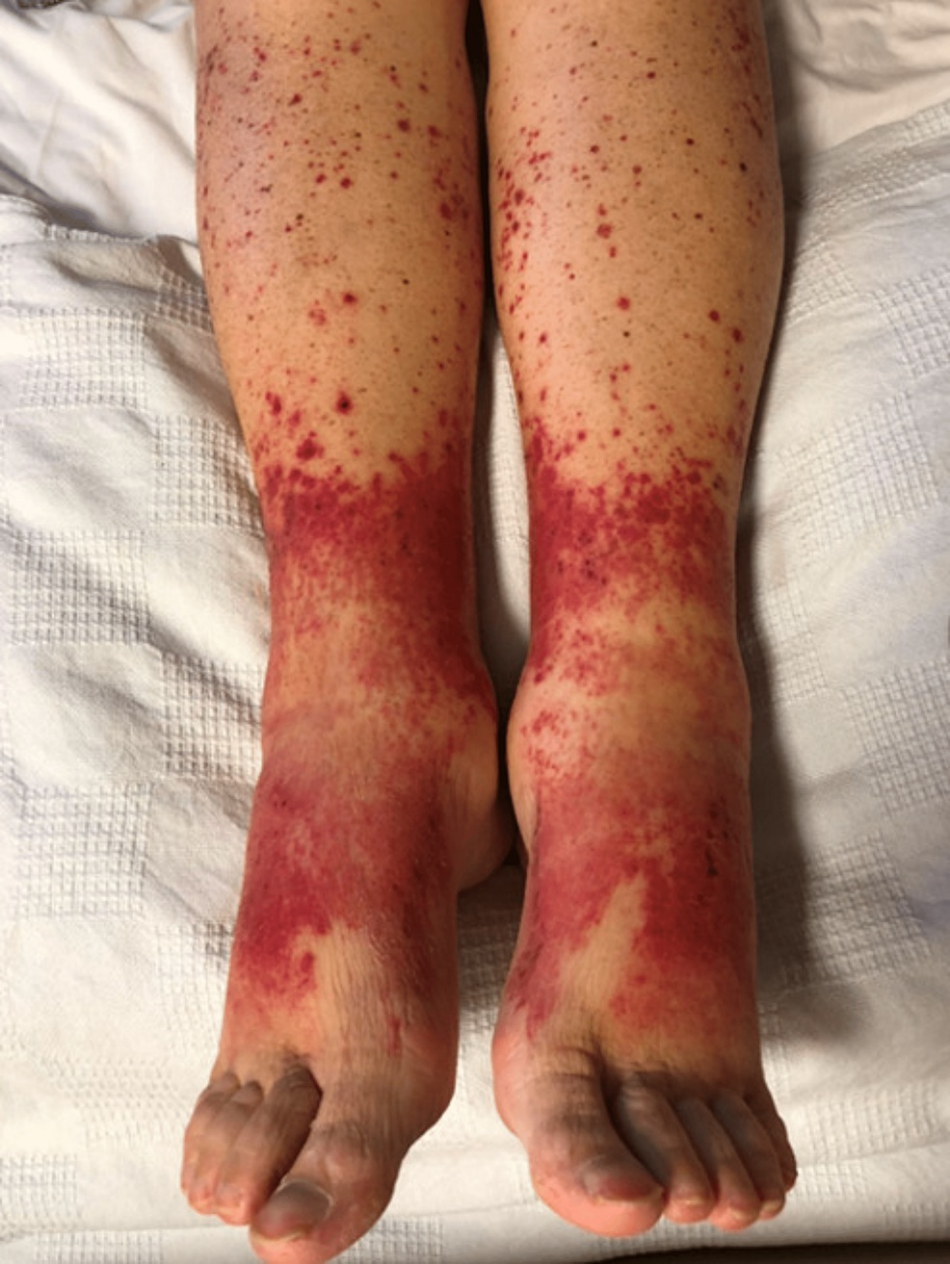
Cryoglobulinemic cutaneous vasculitis.

A petechial rash was appreciated on her lower extremities (Figure 1), and quantitative cryoglobulins (i.e., “cryocrit” for the remainder of the report) was obtained, which was found to be elevated at our cancer hospital. Dermatology was consulted for further recommendations regarding her rash. A skin biopsy was performed revealing leukocytoclastic vasculitis. Her creatinine was elevated on admission to 2.3 mg/dL with heavy proteinuria over 24-hour quantitative measurement (Table 1). Nephrology was consulted as there was a concern for intrinsic renal disease process. A renal biopsy was obtained revealing acute thrombotic microangiopathy, acute tubular injury, and moderate interstitial fibrosis with 30% tubular atrophy. Her blood pressure was controlled with careful adjustment of her antihypertensives. Notable laboratory work obtained during her time in the cancer hospital included C-reactive protein (CRP) of 7.6 mg/L, kappa quantitative free light chains of 86.8 mg/L, lambda quantitative free light chains of 46.3 mg/L (kappa/lambda ratio: 1.88), complement 4 (C4) of 2, rheumatoid factor of 227 IU/mL, hemoglobin negative antineutrophil cytoplasmic antibodies (ANCA), negative double-stranded DNA, and negative hepatitis C antibodies (Table 1).

**Table 1:** Remarkable laboratory work at the cancer hospital. ANA: antinuclear antibody, CRP: C-reactive protein, ANCA: antineutrophil cytoplasmic antibodies, C4: complement 4, dsDNA: anti-double-stranded DNA, IU: international units

Laboratory test	Value/result	Reference range
Quantitative cryoglobulins (cryocrit)	Positive in 24 hours	Negative in 72 hours
ANA	Positive (no titer)	Not detected
CRP	7.6 mg/L	<3 mg/L
Creatinine	2.3 mg/dL	0.5-1 mg/dL
Protein urine, quantitative	2,133 mg/24 hour	40-150 mg/24 hour
ANCA	<1:20	<1:20
Rheumatoid factor	227 IU/mL	0-14 IU/mL
C4	2 mg/dL	10-40 mg/dL
Kappa quantitative free light chains	86.8 mg/L	3.3-19.40 mg/L
Lambda quantitative free light chains	46.3 mg/L	5.71-26.3 mg/L
Kappa/lambda ratio	1.88	0.26-1.65
dsDNA	Negative	Not detected
Hepatitis C antibody	Negative	Not detected

The patient was started on prednisone 40 mg daily to treat her cryoglobulinemia at our cancer hospital. It was decided that it would be in her best interest to be transferred to a nearby large academic general medical center to undergo a formal rheumatologic evaluation. She was continued on prednisone 40 mg daily. Antinuclear antibody (ANA) was retested with a titer of 1:160, and Smith antibody and ribonucleoprotein (RNP) antibody were negative (Table 2). Rheumatoid factor after several days of treatment with prednisone was 128 IU/mL with negative cyclic citrullinated peptide (CCP). CRP was 4.4 mg/dL. Centromere B antibody, RNA polymerase III antibody, cardiolipin antibody, and B2 glycoprotein IgG, IgM, and IgA antibodies were negative. Sjögren’s syndrome-related antigen A (SSA/Ro) antibody was elevated, and SSB/La antibody was negative. On further history-taking during this hospitalization, it was elicited that she had been dealing with chronic dry eyes and mouth for several years even prior to her diagnosis of marginal zone lymphoma. She was diagnosed with presumptive Sjögren’s syndrome, which was favored to be the cause of her cryoglobulinemia as well as her acute renal failure and hypertensive emergency, her leukocytoclastic vasculitis, her anemia, and her hypocomplementemia. In late 5/2019 (almost a month after her initial admission to her cancer hospital), her cryocrit was tested and was negative. She received her first dose of 1 g intravenous rituximab while admitted to the academic teaching center, and a follow-up was scheduled in the rheumatology clinic for ongoing treatment. Her anemia, hypocomplementemia, renal insufficiency, and leukocytoclastic vasculitis improved by the time of her hospital discharge. Her SSA/Ro antibody remained positive on repeat testing in the rheumatology clinic, reinforcing the diagnosis of Sjögren’s syndrome.

**Table 2:** Remarkable laboratory work at the general medical center. ANA: antinuclear antibody, CRP: C-reactive protein, SSA: anti-Sjögren’s syndrome-related antigen A autoantibodies, Ab: antibody, C4: complement 4, RNP Ab: ribonucleoprotein antibodies, RNA polymerase III Ab: anti-RNA polymerase III antibodies, AU or U: arbitrary unit, IU: international unit, GPL: IgG phospholipid unit, SGU: standard IgG beta-2 glycoprotein unit

Laboratory test	Value/result	Reference range
Quantitative cryoglobulins (cryocrit)	Negative	Negative in 72 hours
ANA	Positive (1:160)	Not detected
CRP	4.4 mg/L	<3 mg/L
Creatinine	2.3 mg/dL	0.5-1 mg/dL
SSA/Ro Ab	200 AU/mL	0-40 AU/mL
SSB/La Ab	3 AU/mL	0-40 AU/mL
Rheumatoid factor	128 IU/mL	0-14 IU/mL
C4	<2.9 mg/dL	10-40 mg/dL
Smith Ab	0 AU/mL	0-40 AU/mL
RNP Ab	2 AU/mL	0-40 AU/mL
Centromere B Ab	Negative	<29 AU/mL
RNA polymerase III Ab	Negative	Negative
Cardiolipin IgG Ab	2 GPL	0-12 GPL
B2 glycoprotein Ab	0 SGU	0-20 SGU

## Discussion

Cryoglobulins are immunoglobulins and complements or immunoglobulins alone that precipitate in plasma at temperatures less than 37°C. They deposit in medium- and large-sized vessels, leading to endothelial and end-organ damage. According to Brouet criteria, cryoglobulinemia is categorized into three subgroups based on its immunoglobulin composition. Type I has isolated monoclonal immunoglobulins, such as IgG or IgM, and is seen in the setting of hematologic disorders such as multiple myeloma, Waldenstrom macroglobulinemia, monoclonal gammopathy of undetermined significance (MGUS), or chronic lymphocytic leukemia (CLL). Type II has monoclonal immunoglobulins with rheumatoid factor activity and polyclonal immunoglobulins, associated with autoimmune diseases such as Sjögren’s syndrome, malignancy, or infections, particularly hepatitis C. Hepatitis C has also been proven to be associated with the development of marginal zone lymphoma in the literature [4]. Type III cryoglobulinemia has polyclonal immunoglobulins of various types (such as IgG and IgM). Types II and III are referred to as mixed cryoglobulinemias because they consist of both IgG and IgM components [5]. Our patient likely had type II cryoglobulinemia (as above, the centers did not analyze the specific type) as most cryoglobulinemia in systemic autoimmune disease is type II.

Cutaneous vasculitis is probably the most characteristic manifestation of cryoglobulinemic vasculitis, as demonstrated in our patient. About 20% of patients with cryoglobulinemia present with nephropathy at diagnosis, and about 30% have renal complications during the disease course. Renal features noted in cryoglobulinemia include proteinuria, microscopic hematuria, red blood cell casts, and renal failure [6]. In primary Sjögren’s syndrome, cryoglobulinemia is associated with extraglandular involvement, with an increased risk for developing B-cell lymphoma, primarily low-grade lymphomas, with MZL being the most common histologic subtype. Patients with primary Sjögren’s syndrome are up to 10-44 times more likely to develop these lymphomas than healthy controls [7]. Interestingly, our patient’s malignancy was not thought to have played a major role in the development of her cryoglobulinemia given that her bone marrow was without residual activity. However, patients such as our patient should be closely monitored for the development of B-cell lymphoma in the future. It does not seem that there have been major case studies to explore the link between MZL and cryoglobulinemia, but some interesting case reports do exist. One such case reviews a female subject in her 60s with an extranodal small intestinal mucosa-associated lymphoid tissue (MALT) tumor histologically identified as MZL with monoclonal (IgM kappa type) cryoglobulinemia presenting with a similar rash to our subject, proliferative glomerulonephritis, and nephrotic syndrome. This patient was found to have ascites that when studied revealed cells consistent with lymphomatous spread [8]. Another case of a 78-year-old male with a MALT tumor of the small intestine (also MZL) was found to have type I cryoglobulinemia (again IgM kappa) with minor involvement of the bone marrow [9]. To reiterate, our patient had no such signs of MZL recurrence or hepatitis C.

Treatment of cryoglobulinemia is typically aimed at the underlying cause, which in our case was immunosuppression with steroids and rituximab aimed at the patient’s Sjögren’s syndrome. Importantly, there are no well-delineated guidelines yet for the treatment of cryoglobulinemia, and thus, case reports/series remain an important pillar in the understanding of this disease.

## Conclusions

This case illustrates a presumptive case of mixed cryoglobulinemia in a patient with a history of marginal zone lymphoma in remission and a recent diagnosis of Sjögren’s syndrome. Although we cannot be certain of the exact nature of her cryoglobulinemia, nor can we completely eliminate whether her MZL may have contributed to its development, the diagnosis of Sjögren’s syndrome and the complex nature of her presentation highlight the challenges that occur at the point of care.
